# Vibrotactile beat sensitivity in deaf individuals: behavioral and neural evidence

**DOI:** 10.1093/cercor/bhag111

**Published:** 2026-07-30

**Authors:** Sean A Gilmore, Phuong-Nghi Pham, Frank A Russo

**Affiliations:** Department of Psychology, Toronto Metropolitan University, Toronto, ON, Canada; Department of Psychology, Toronto Metropolitan University, Toronto, ON, Canada; Department of Psychology, Toronto Metropolitan University, Toronto, ON, Canada

**Keywords:** beat sensitivity, deaf, EEG, sensorimotor synchronization, vibrotactile

## Abstract

Beat sensitivity is typically studied in the auditory modality, yet the temporal prediction mechanisms that support beat processing are thought to be supramodal and may be accessible through other sensory channels. Deafness provides a powerful model for examining whether these mechanisms can be recruited through somatosensory input. We compared vibrotactile beat sensitivity in deaf (*n* = 17) and hearing (*n* = 16) adults using frequency-tagging EEG and sensorimotor synchronization (SMS) during exposure to isochronous and temporally complex vibrotactile rhythms. Neural entrainment was quantified as spectral power at beat-related frequencies, while SMS variability indexed behavioral precision. Deaf participants exhibited significantly stronger neural entrainment to vibrotactile rhythms than hearing controls. Behaviorally, deaf individuals showed reduced tapping variability, an effect that was selectively enhanced for temporally complex rhythms, suggesting more efficient extraction of higher-order temporal structure. These neural and behavioral enhancements occurred despite comparable within-group entrainment across rhythm types. Together, these findings demonstrate that beat-based temporal prediction can be robustly achieved through vibrotactile input in the absence of auditory experience. This work provides combined behavioral and neural evidence for vibrotactile beat sensitivity in deaf individuals and indicates that somatosensory pathways can support predictive timing mechanisms typically associated with audition.

## Introduction

Understanding how the brain extracts temporal regularities from sensory input is central to theories of predictive coding, rhythmic processing, and sensorimotor integration. Although audition is typically the dominant channel for temporal prediction in music, neural systems supporting beat sensitivity are not inherently modality-specific and may be accessible through alternative sensory pathways. Early deafness provides a powerful model for testing the extent to which temporal prediction mechanisms, including those typically engaged by auditory rhythms, can be recruited through non-auditory modalities such as vision and touch.

## Temporal processing and beat sensitivity

Musical rhythm depends on detecting temporal regularities that support predictive timing. Beat *sensitivity* is the capacity to extract, anticipate, and align with a periodic pulse and is thought to be supported by a distributed network involving auditory cortex, basal ganglia, SMA, premotor cortex, and cerebellum (Grahn and Brett 2007; Chen et al. 2008; Grahn and Rowe 2009). fMRI studies show that this network engages not only for explicit beats but also for rhythms requiring internally generated periodicity, underscoring its central role in predictive timing and hierarchical temporal structure.

## Cross-modal recruitment in deaf individuals

In individuals with early deafness, auditory cortices undergo substantial cross-modal reorganization yet retain sensitivity to temporal structure (Bola et al. 2017). Somatosensory and visual inputs reliably activate superior temporal regions and dorsal-stream sensorimotor pathways (Auer et al. 2007; Karns et al. 2012; Bola et al. 2017), areas strongly implicated in beat sensitivity in hearing individuals. This suggests that temporal prediction mechanisms normally accessed through audition may remain functionally available through other sensory modalities.

## Beat sensitivity without audition: what is known

Only two studies have directly examined beat sensitivity in deaf participants. Iversen et al. (2015) used visual rhythms and found that deaf individuals showed superior synchronization to discrete visual onsets and more anticipatory tapping phases, behavioral markers consistent with efficient internally generated temporal predictions. In contrast, Tranchant et al. (2017) reported no group differences in vibrotactile synchronization, but interpretation is limited because of a very small deaf sample (*n* = 7) and the use of a single rhythm type without manipulation of temporal complexity. Moreover, the study relied exclusively on behavioral measures, thereby limiting interpretation regarding the neural mechanisms underlying potential group differences. These methodological constraints leave open the question of whether deaf individuals show robust vibrotactile beat sensitivity under conditions that preserve somatosensory acuity and appropriately challenge temporal prediction mechanisms.

## Non-auditory pathways for temporal processing

Evidence from neuroimaging shows that vibrotactile input engages the same predictive timing network implicated in auditory beat sensitivity. fMRI work demonstrates that vibrotactile stimulation activates premotor cortex, SMA, and posterior superior temporal cortex (Araneda et al. 2017), consistent with a modality-general mechanism for encoding low-frequency temporal structure. Behavioral evidence further indicates that vibrotactile stimulation can directly enhance rhythmic engagement. In hearing individuals, coupling auditory music with tactile stimulation, particularly at low frequencies, leads to more forceful tapping, increased spontaneous body movement, and higher perceived groove and enjoyment, suggesting a tight functional coupling between tactile and motor systems during rhythm processing (Hove et al. 2020).

To date, two studies have examined vibrotactile beat sensitivity in hearing populations but with different emphases. Ammirante et al. (2016) focused exclusively on sensorimotor synchronization (SMS) and showed that while synchronization to isochronous vibrotactile rhythms is comparable to auditory rhythms, performance deteriorates markedly for non-isochronous patterns. Gilmore and Russo (2021) extended this work by combining SMS with frequency-tagging EEG, showing not only reduced synchronization accuracy for vibrotactile rhythms compared to auditory rhythms but also weaker (but still significant) neural entrainment at beat-related frequencies. Notably, Gilmore and Russo (2021) was the first empirical investigation of vibrotactile beat sensitivity, inclusive of rhythmic neural entrainment and SMS.

## The frequency-tagging debate

Although frequency-tagging has become a central tool for assessing neural entrainment in beat perception, its interpretation is not without controversy. Critics argue that increases in spectral amplitude at beat-related frequencies may reflect the summation of stochastic evoked responses rather than genuine oscillatory synchronization (Capilla et al. 2011; Novembre and Iannetti 2018). However, simulations by Doelling et al. (2019) showed that evoked models produce rate-dependent phase lag and poorly predict MEG responses during rhythmic listening, whereas oscillatory models exhibit stable phase relationships and superior predictive accuracy. While evoked activity likely contributes to some components of the response, the convergence of behavioral, modeling, and neuroimaging evidence supports a substantive role for oscillatory mechanisms in temporal prediction.

## Critical gap

Despite robust evidence that vibrotactile rhythms can engage predictive timing networks in hearing individuals, it remains unknown whether these mechanisms operate similarly—or perhaps more efficiently—in individuals who lack auditory experience. The existing studies on deaf participants provide mixed results, rely solely on behavioral indices, and lack the methodological precision needed to assess the neural mechanisms underlying beat sensitivity. Crucially, no study has examined vibrotactile beat sensitivity in deaf individuals using frequency-tagging EEG, leaving unresolved whether oscillatory mechanisms implicated in beat sensitivity remain accessible through somatosensory channels in the absence of audition.

## Goal of the present study

The present study addresses this gap by partially replicating and extending Tranchant et al. (2017) using (i) a larger deaf sample, (ii) vibrotactile rhythms that vary in temporal complexity, (iii) stimulation delivered to non-glabrous skin to avoid sensitivity decline, and (iv) frequency-tagging EEG to quantify neural entrainment. This design enables us to test whether deaf individuals exhibit enhanced, reduced, or comparable vibrotactile beat sensitivity relative to hearing individuals, and to characterize the neural mechanisms supporting temporal prediction in the absence of auditory input.

## Methods

This study was approved by the Research Ethics Board of Toronto Metropolitan University (REB protocol #2019-101). All participants provided informed consent prior to participation. Consent procedures and study materials were provided in accessible formats appropriate to participants’ communication preferences.

## Participants

Based on a sample size calculation using Gpower, we determined that using a mixed-effect multiple regression with 2 predictor variables; auditory access (deaf vs. hearing); temporal complexity (isochronous vs. house rhythm); and an interaction between the two (auditory access * temporal complexity). With an expected power of 0.8, we determined a total sample size of 40 individuals (20 hearing and 20 deaf). Our initial inclusion criteria for the deaf group were congenital deafness, although due to recruitment constraints, we ultimately broadened this to participants who identify as deaf and who experienced profound hearing loss as late as adolescence. This resulted in a heterogeneous deaf sample comprising congenitally deaf and late-deafened individuals with varying degrees of hearing loss. We excluded individuals who were CI users or those who regularly use hearing aids and an additional exclusion criterion in both samples was the presence of any neurological disorders (ie epilepsy, stroke, traumatic brain injury, etc.).

In total we tested 40 participants. Seven participants were rejected (3 deaf and 4 hearing) due to technical issues, with either the EEG system or the hardware-software system for recording SMS, which led to an incomplete testing session. The final sample consisted of 33 participants (17 deaf and 16 hearing).

The deaf group included 9 female and 8 male participants and had a median age of 19 years (M = 21.31, SD = 14.90). Nine participants were congenitally deaf, 5 were late-deafened with profound hearing loss, and 2 were late-deafened with moderate hearing loss; hearing status was unspecified for 1 participant. Four participants reported musical training, with an average of 4.41 years (SD = 1.83). The hearing group included 11 female and 3 male participants; 2 participants did not specify their sex. The median age was 22 years (M = 24.66, SD = 7.16). Seven participants reported musical training, with an average of 3.63 years (SD = 2.53).

## Stimuli

**Figure 1:**
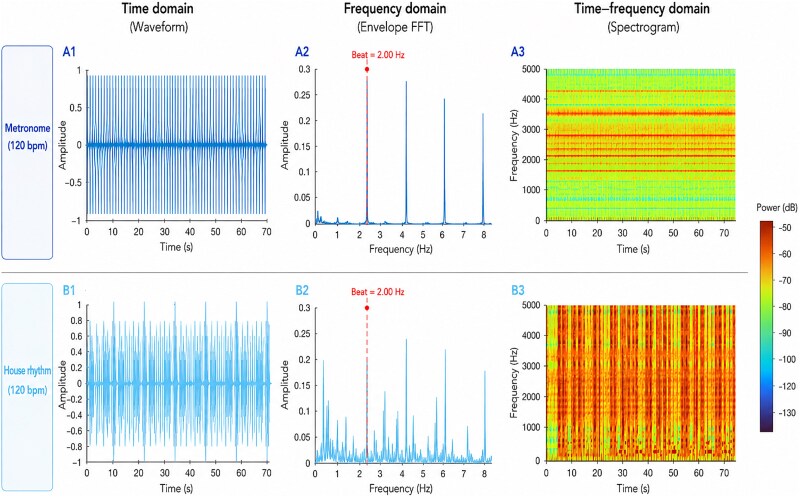
(A1–A3) Metronome (120 BPM). (B1–B3) House rhythm (120 BPM). (A1, B1) Time-domain waveforms. (A2, B2) Envelope FFT showing peaks at the beat frequency (2 Hz; dashed line) and harmonics; the metronome shows discrete periodic peaks, whereas the house rhythm shows broader spectral energy. (A3, B3) Spectrograms (0–5000 Hz; 25-ms window, 20-ms overlap, dB scale), illustrating regular temporal energy in the metronome and more variable, broadband energy in the house rhythm.

Stimuli consisted of a metronome and a house rhythm (adapted from Tranchant et al. 2017). The metronome consisted of a series of equally spaced kick drums taken from the house rhythms, generating a metronomic pulse. The house rhythm was constructed of digitally generated drum sounds (ie kick, snare and high hat). These rhythms consisted of an isochronous pulse from a kick drum and additional snare and high-hat events at metric subdivisions of the kick drum (see Figure 1).

Similar to the metronome, the house rhythms had an isochronous pulse (ie kick drum) providing a cue to the beat (see Figure 1: B2); they were more temporally complex given the events at subdivisions of the isochronous pulse. Metronome and house rhythms were played at three different tempi (110 bpm, 115 bpm and 120 bpm) to reduce any practice effects that may have emerged across through repeated exposure to a specific tempo. This gave us six unique rhythmic stimuli (2 rhythms × 3 tempi).

**Figure 2:**
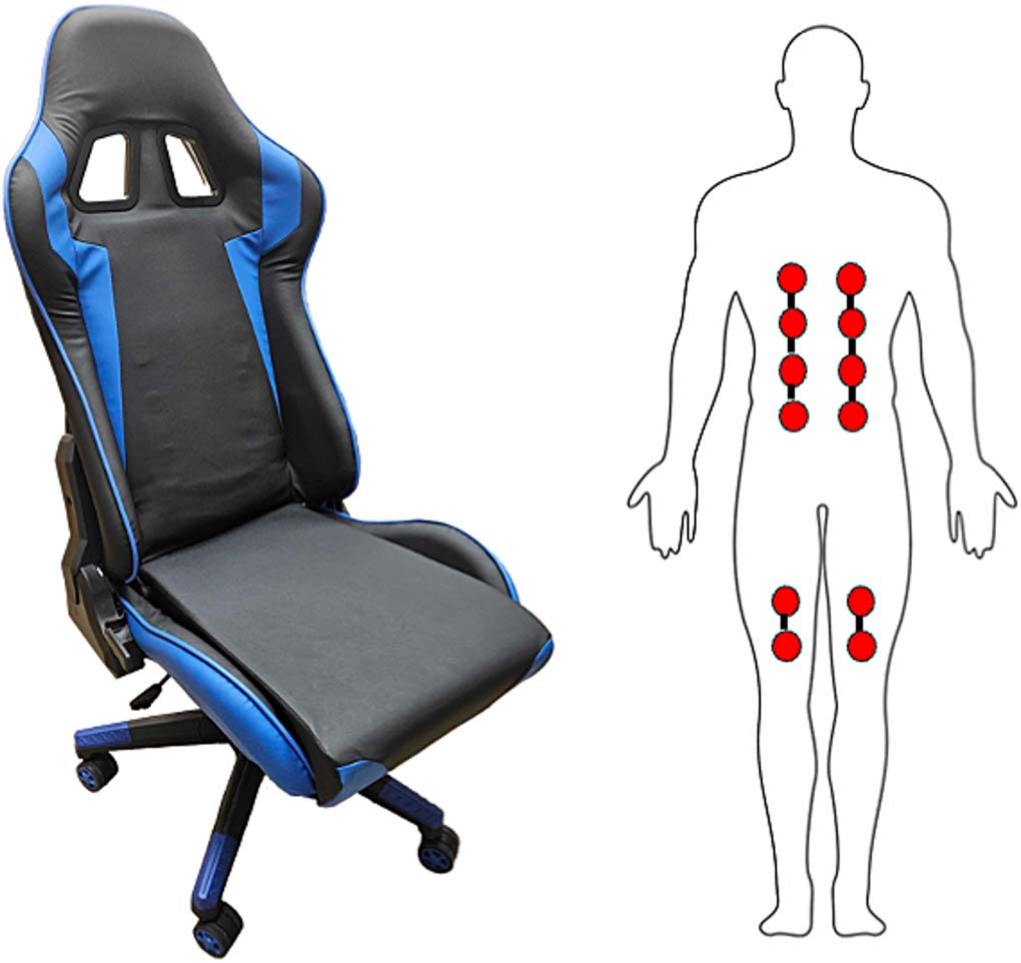
A figure of the ImmersX Vibro-Acoustic Chair with the approximate anatomical locations of vibrotactile stimulation roughly located along bi-lateral thoracic and lower thigh regions.

Each rhythm was preceded with an 8-beat metronome corresponding to the tempo of the ensuing rhythm (adopted from Iversen et al. 2005; Ammirante et al. 2016; Gilmore and Russo 2021). The presentation order of the rhythms was counterbalanced between participants, and the order of the tempi was randomized. Each rhythm was presented once. Stimuli were presented on the ImmersX Vibro-Acoustic Chair (See Figure 2). This chair converts auditory signals into vibrotactile experiences through an array of 6 × 2 transducers located along the back and bottom of the chair roughly placed on thoracic (8 transducers) and lower thigh (4 transducers) regions. Vibration intensity associated with the house rhythm and metronome were quantified using a Larson Davis HVM100 vibration meter with Fa (flat) frequency weighting. Measurements were obtained across a frequency range of 0.4 to 1260 Hz. The maximum instantaneous weighted acceleration (peak) for the house rhythm reached 0.463 m/s^2^, with a maximum peak amplitude (Amp) of 0.557 m/s^2^ recorded over the duration of the stimulus. The maximum instantaneous weighted acceleration (peak) for the metronome reached 0.463 m/s^2^, with a maximum peak amplitude (Amp) of 0.51 m/s^2^ recorded over the duration of the stimulus.

## Procedure

Once consent was obtained, an overview of the experiment was provided using a short video which contained instructions in written English and American-Sign Language. Following this overview, a series of questionnaires was administered concerning demographics, music background and sign language proficiency. After questionnaires were completed, participants were brought into the sound-attenuated booth and asked to sit on the ImmersX Vibro-Acoustic Chair.

All participants were outfitted with insert earphones (3M E-A-RTONE GOLD). To mitigate perceivable sounds from the vibrotactile chair, hearing participants conducted a white noise calibration task. For this task, hearing participants heard white noise while the vibrotactile chair played a 4-beat metronome, during which participants were asked to adjust the level of white noise until it completely masked the sound emitted from the vibrotactile chair. White noise levels were measured as A-weighted equivalent continuous levels (LAeq) over 10-min intervals using a calibrated sound level meter. Average levels of white noise for hearing participants was recorded as LAeq,10 min = 70.1 dB(A) (SD = 1.21). This individually calibrated level of white noise was presented during the entire duration of the stimuli. For deaf individuals, white noise was presented at a fixed rate (55 dB LAeq), based on pilot testing with deaf individuals. Before starting the tasks, all participants watched a video which explained the difference between a rhythm and a beat. This was preceded by a practice trial, where participants were asked to tap along to excerpts of the rhythms. This was supervised by a researcher to ensure correct understanding and perception of the beat. Following this, participants either started the passive or tapping task. The order of passive and tapping tasks were counterbalanced. All tasks were presented using PsychToolbox (Brainard 1997), a MATLAB package designed for psychological experimentation.

### Passive task

For the passive task each participant was outfitted with a Biosemi EEG system with 6 external channels. The Biosemi EEG system used for data collection was a 128-channel active electrode system recorded at 512 Hz sample rate. The 6 external electrodes were placed on bi-laterally on mastoids, lateral junction of the upper and lower eyelids and orbital ridge. Once the EEG system was secured, impedances and noisy channels were fixed such that all 128-channels were within +/− 40 mV DC offset.

After this, participants started the passive task. The task began with instructional videos which reminded the participant of the difference between the rhythm and beat and instructed them to sit still and reduce movement during the presentation of each rhythm. Following these instructions, participants experienced each rhythm at a self-directed pace, wherein participants had pauses after each trial followed by a prompt “press any key to start the next trial”. EEG signals were recorded using ActiView software on a computer outside of the booth. The duration of the passive task was roughly 40 min including the EEG capping.

### Tapping task

For the tapping task, participants first watched an instructional video. This instructed participants to tap along to the beat of each rhythm. Taps were recorded on a Roland Handsonic drum pad which sent a MIDI signal to a digital audio workstation (ie Garageband). During tapping trials, a copy of the stimulus was simultaneously recorded alongside the MIDI signal. This allowed us to time lock the MIDI signal to onset of the beat in the stimulus for later analysis of SMS. The drum pad was placed at arm’s reach from participants. Through an instructional video, participants were instructed not to switch which hand they tapped with and to make sure their taps were firm, but not too hard as to cause discomfort. The duration of the tapping task was roughly 15 min.

## Pre-processing and frequency tagging analysis

### Preprocessing

All signal processing of EEG data was done in EEGLAB (Delorme and Makeig 2004). Raw EEG data first underwent preprocessing which entailed: high pass filtering (0.1 Hz); rejection of noisy channels using “cleanArtifacts” function (Kothe and Makeig 2012) and then interpolation of rejected channels. Following this an infomax independent component analysis (ICA) was computed. Artifactual components (eg eyeblinks) were flagged and removed using the ADJUST toolbox (Mognon et al. 2011), which automatically detects and removes artifactual components based on spatial and temporal features. Cleaned data was then parsed into 55 s epochs. Each epoch started 1 s after the beginning of each trial to avoid a transient auditory response (Saupe et al. 2009) and roughly 3 s before the end of each trail to avoid overlap between trials.

### Frequency-tagging

Epoched EEG data was used to calculate whole brain measures of neural entrainment following previous methods (Nozaradan et al. 2011; Nozaradan et al. 2012; Gilmore and Russo 2021). For each electrode, epoched signals underwent a Fourier transformation. To isolate the induced activity from background noise (eg muscle movement, spontaneous neural responses, etc.) a noise-floor subtraction technique was used and from each bin of the magnitude spectra, the average amplitude across 4 flanking bins (2 on each side of the given bin) was subtracted. This produces a magnitude spectrum for each electrode, from which neural entrainment for each rhythm condition was calculated as mean power across 3 frequency bins centered on the beat frequency (relative to each tempi) of each rhythm (1 centered on the beat frequency and 2 flanking bins). Neural entrainment values were averaged across electrodes, yielding a whole brain measure of entrainment. Neural entrainment at harmonic frequencies of the beat frequency (2× and 3×) was also calculated (after Gilmore and Russo 2021).

### Tapping data

MIDI files from each participant were epoched into 6 single trial MIDI recordings. MIDI recordings for each participant were analyzed using *MIDI Toolbox 1.1* (Toiviainen and Eerola 2016), a MATLAB based toolbox which provides timing metrics for each event of a MIDI recording. This toolbox produces a series of the onsets (s) for each MIDI event (ie tap), relative to the start of the trials. Intertap Interval (ITI) was calculated as time between onsets. To eliminate double taps and small breaks during a trial, ITIs that were larger than +/− 2 standard deviations were rejected.

**Figure 3:**
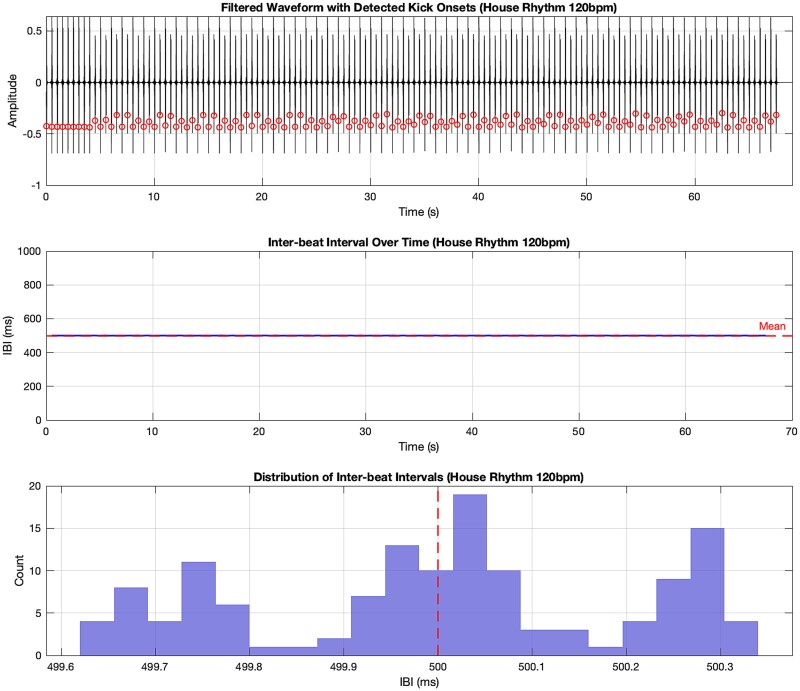
Analysis of kick drum timing in the 120 BPM house rhythm stimulus. The first panel shows the waveform after bandpass filtering between 30 and 130 Hz to isolate kick drum events, with detected onset peaks marked. The second panel plots the inter-beat interval (IBI) across successive kick drum onsets over time. The third panel shows the distribution of IBIs. The distribution is centered around 500 ms (corresponding to 120 BPM) with approximately ±3 ms variability, indicating highly stable rhythmic timing throughout the stimulus.

We then calculated a grid of equally spaced beat positions for each trial based on the relative tempo (eg 120 bpm = 500 ms) of each stimulus. For metronome trials the pulse of the beat corresponds to the onset of each event, however, we wanted to ensure accuracy of the beat grid for House rhythms, given that they contain more events at different hierarchies of the beat. To do this, we first applied a bandpass filter (30 to 130hz) to isolate the kick drum. We then calculated the interbeat interval by using the *findpeaks* function in MATLAB to determine the onset of each kick drum event. From these onsets, we calculated the inter-onset/beat interval. As shown in Figure 3, the kickdrum followed a consistent pulse across time (+/− .3 ms) which corresponds to the tempo (eg 500 ms for 120 bpm). Thus, the beat grid for each House rhythm was defined by its tempo.

The onsets of non-rejected taps were aligned to the nearest beat in the beat grid, and asynchrony was computed as the temporal difference between each tap and its closest beat. Tapping variability, a standard index of SMS ability (eg Patel et al. 2005; Ammirante et al. 2016; Gilmore and Russo 2021), was defined as the linear standard deviation of these asynchronies within each trial. Lower values indicate more stable synchronization.

### Statistical analysis

Data was modeled using the *lmer4* package (Bates et al. 2015) in R using a Satterwaite method to estimate degrees of freedom. Two mixed-effect multiple linear regressions were used to predict enhancements in neural entrainment and SMS. Fixed-effects in the model were Group (hearing vs. deaf), Rhythm Complexity (metronome vs. house) and an interaction term between the two. To examine the relationship between behavioral and neural indices of rhythm processing, an additional linear mixed-effects model was conducted in which neural entrainment was modeled as a function of tapping variability (SMS variability), Group (hearing vs. deaf), and their interaction. For this model, tapping variability was mean-centered to facilitate interpretation of lower-order effects in the presence of the interaction. This model tested whether the association between tapping variability and entrainment differed as a function of auditory status.

Given the presence of both between and within- and between-subject factors, observations were not independent, and covariance within participants was expected. To account for this, mixed-effects models were specified with participant ID included as a random intercept. Fixed effects were evaluated using dummy coding, with the hearing group as the reference level for Group and metronome rhythms as the reference level for Rhythm Complexity.

Before modeling, entrainment values were visually inspected to identify extreme values likely to be artifactual (eg disproportionately elevated spectral power relative to neighboring frequency bins). This step was used to remove clear non-physiological anomalies that are not well captured by automated criteria. Following this initial screening, outliers were identified using a boxplot criterion (±1.5 × IQR) within each group, providing a standardized and reproducible method for detecting statistically extreme observations. For the neural entrainment data, ~11% and ~7% of trials were flagged as outliers and rejected in the deaf and hearing groups, respectively.

Tapping variability (SD of asynchrony) exhibited positive skew, as is typical for variability measures. In contrast to the neural entrainment data where extreme values were treated as artifacts and excluded, extreme values in tapping variability are expected to reflect genuine behavioral variation (Repp and Su 2013). Accordingly, to improve adherence to model assumptions while preserving the full range of performance, following Cannon et al. (2024), values were log-transformed prior to analysis, stabilizing variance and reducing the influence of extreme observations.

Assumptions of normality for the final model were assessed through a visual inspection of model residuals. Based on this, assumptions of normality were met for all models. Fixed-effects for all models are reported on in the results section. For all fixed-effects threshold for the rejection of the null hypothesis was set at the alpha level of 0.05.

## Results

### Neural entrainment

The fixed effects in the mixed-effects model revealed a significant effect of group, *b* = 0.018, 95% CI [0.0009 0.0349], *t*(50) = 2.09, *P* = 0.04. Compared to the hearing group, levels of neural entrainment in the deaf group increased by 0.018 mV when collapsing across rhythm complexity (Figure 4a). This group difference is also evident in the descriptive spectral profiles (Figure 4b), where larger amplitude peaks at beat-related frequencies can be observed in the deaf group across conditions. The main effect of rhythm complexity was not significant, *b* = −0.0004, 95% CI [−0.0124 0.0114], *t*(142) = −0.07, *P* = 0.93. The interaction between group and rhythm was also not significant, *b* = −0.00007, 95% CI [−0.017 0.017], *t*(142) = −0.008, *P* = 0.99.

**Figure 4a:**
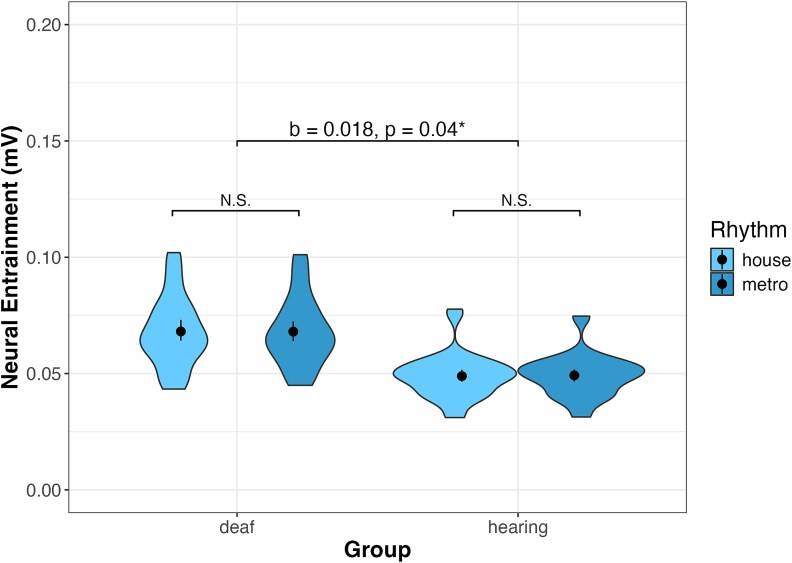
Neural entrainment in deaf and hearing groups across rhythms. Neural entrainment values are based on predicted values from fixed effects of the mixed-effect model (entrainment ~ group * rhythm + (1|ID)) output. Error bars reflect bootstrapped confidence 95% intervals of fixed-effect estimates. ^*^P < 0.05, ^**^P < 0.01, ^***^P < 0.001, N.S non-significant.

**Figure 4b:**
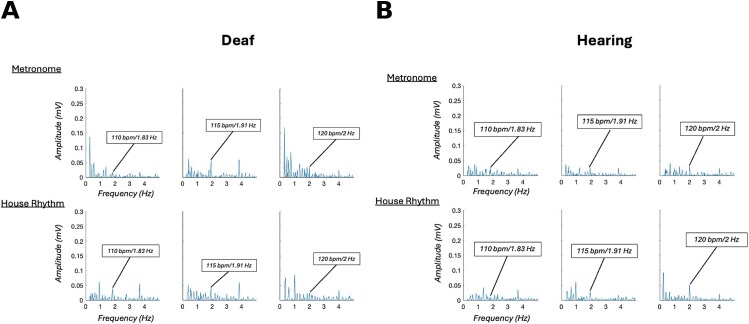
Average frequency spectra plots across rhythm complexity and tempi for deaf (A) and hearing (B) groups. The EEG amplitude spectrum (mV) between 0 and 5 Hz averaged across electrodes following the noise subtraction technique (see methods). The left depicts the amplitude spectra of the deaf group and the right depicts the amplitude spectra of the hearing group. Larger amplitude spikes can be seen at beat frequencies in the deaf group compared to the hearing group.

### Sensorimotor synchronization

**Figure 5:**
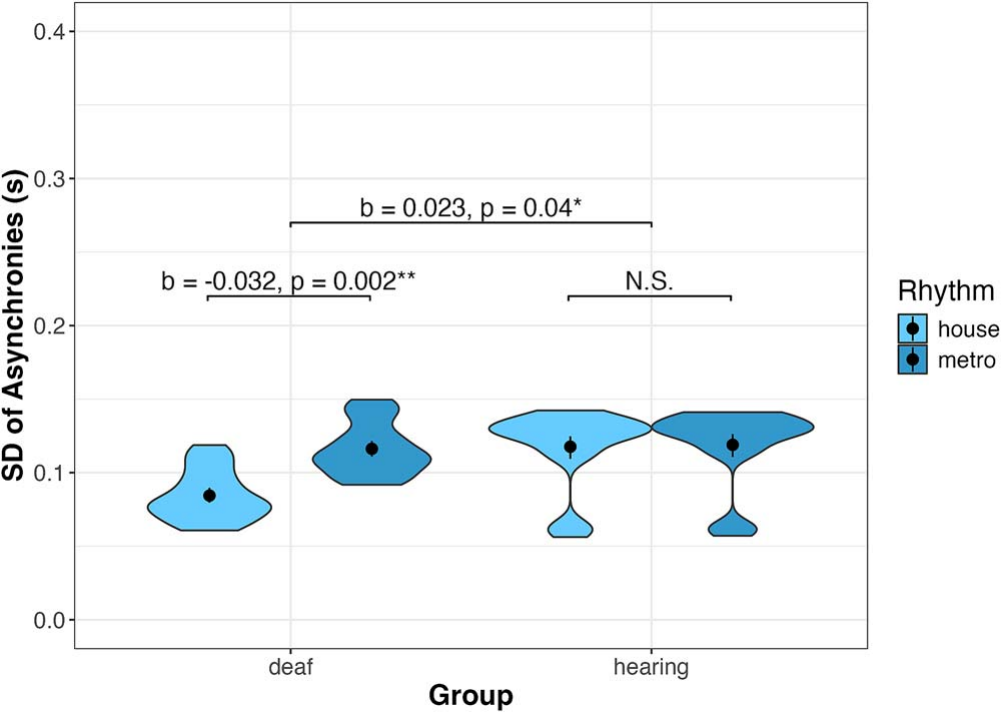
Sensorimotor synchronization (SMS) between deaf and hearing groups across rhythms as measured by the standard deviation of asynchronies. The plotted SD of asynchronies are predicted values obtained from the fixed effects of the mixed-effect model (variability ~ group * rhythm + (1|ID)). Error bars reflect bootstrapped confidence 95% intervals of fixed-effect estimates. ^*^P < 0.05, ^**^P < 0.01, ^***^P < 0.001, N.S non-significant.

The fixed effects in the mixed-effects model revealed a significant effect of group, *b* = −0.23, 95% CI [−0.444–0.012], *t*(125) = −2.09, *P* = 0.04. Compared to the hearing group, the deaf group showed a significant decrease of 0.023 ms in variability when collapsing across all rhythms. This main effect was qualified by a significant interaction term, *b* = −0.45, 95% CI [−0.869–0.028], *t*(130) = −2.1, *P* = 0.03. Further inspection of the simple slopes (see Figure 5) revealed a significant difference between rhythms for the deaf group, *b* = −0.46, 95% CI [−0.753–0.175], *t*(129) = −3.14, *P* = 0.002, but not for the hearing group, *b* = −0.01, 95% CI [−0.32 0.288] *t*(129) = −0.1, *P* = 0.92. This suggests that deaf individuals showed a greater reduction in tapping variability (0.032 ms.) when tapping to the house rhythm compared to the metronome, whereas no differences between rhythms were revealed in the hearing group.

### Neural entrainment x sensorimotor synchronization

**Figure 6:**
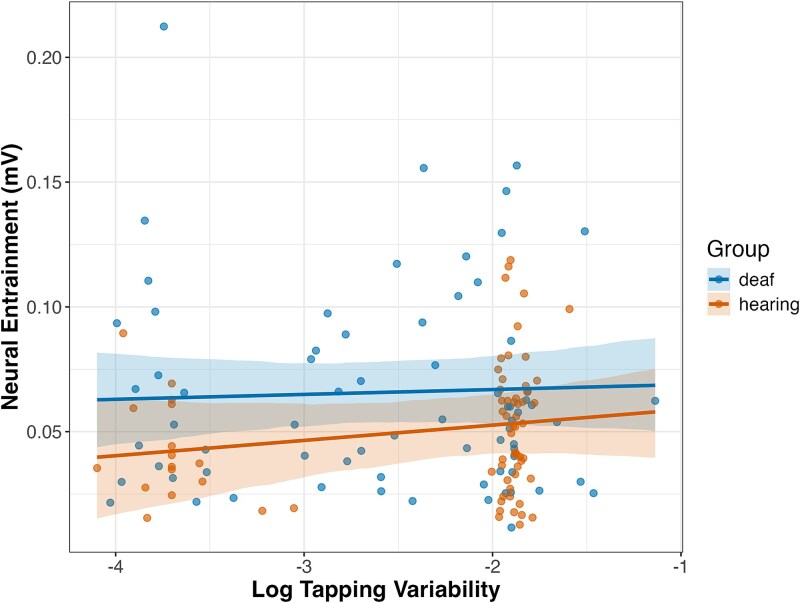
Scatter plot showing the relationship between neural entrainment and sensorimotor synchronization (SMS; indexed by tapping variability) in deaf and hearing groups. Points represent observed participant data, while lines indicate model-predicted values from a linear mixed-effects model including centered log-transformed tapping variability, group, and their interaction, with a random intercept for participant. Shaded regions represent 95% bootstrapped confidence intervals around the fixed-effect estimates. Although neural entrainment was higher in the deaf group, tapping variability did not significantly predict entrainment, and the relationship between tapping variability and entrainment did not differ between groups.

The results of the mixed-effect model (see Figure 6) revealed no significant relationship between tapping variability and neural entrainment, *b* = 0.006, 95% CI [−0.004 0.0158] *t*(138) = 1.1, *P* = 0.27. There was a significant main-effect of Group, *b* = 0.014, 95% CI [0.003 0.025] *t*(138) = 2.52, *P* = 0.012, suggesting greater neural entrainment in the deaf compared to the hearing group. The interaction term between tapping variability and Group was not significant, *b* = −0.008, 95% CI [−0.022 0.005] *t*(138) = −1.16, *P* = 0.25. Overall, this suggests that although neural entrainment was shown to be higher overall in the deaf group, neural entrainment was not predicted by tapping variability and the non-significant interaction indicates that the relationship between tapping variability and neural entrainment did not differ as a function of auditory status.

## Discussion

The aim of this study was to replicate and extend the results of Tranchant et al. (2017) through the stimulation of non-glabrous skin as well as adding additional measures of neural entrainment to more thoroughly measure differences in vibrotactile beat sensitivity between deaf and hearing individuals. Our results suggest that regardless of rhythmic complexity, deaf participants showed higher levels of neural entrainment and more precise SMS compared to hearing controls. Furthermore, pronounced beat sensitivity to house rhythms was observed in the deaf sample, indexed by enhanced SMS that was not evident in the hearing group. Together, these findings indicate that vibrotactile beat-based perception of rhythm may be enhanced deaf individuals.

## Neural entrainment to vibrotactile beats

At the neural level, on average deaf individuals exhibited larger amplitude peaks at beat frequencies than hearing participants (Figure 2a and b). Within the frequency-tagging framework, such peaks are typically interpreted as reflecting entrained low-frequency neural oscillations that phase-lock to the temporal structure of the stimulus (Nozaradan et al. 2011; Duecker et al. 2024). Furthermore, grounded in dynamic attending theory (Jones and Boltz 1989; Large and Jones 1999), low-frequency oscillations have been proposed as a biological mechanism for temporal predictions found in musical beat perception (Large et al. 2015; Henry et al. 2017). Given our results, we can interpret the observed larger amplitude spikes at beat frequencies in the deaf group as an index of refined neural tracking of vibrotactile rhythmic stimuli. As such, our results provide the first empirical evidence of heightened neural tracking of vibrotactile rhythms in deaf individuals.

We note that, despite these group differences, we did not observe a main effect of rhythm type on beat-frequency responses. This factor was included on an exploratory basis, as prior work does not provide a clear prediction regarding whether neural tracking at the beat frequency should be stronger for isochronous versus more complex rhythmic patterns. On one hand, isochronous (metronome-like) rhythms provide highly regular acoustic input that can drive robust, stimulus-locked responses. On the other hand, more complex rhythms, including syncopated patterns, are thought to engage internally generated temporal predictions to a greater extent, potentially strengthening beat representation through top-down mechanisms (eg predictive processing accounts; see Vuust et al. 2018). These competing influences of stimulus-driven regularity versus internally generated prediction make it difficult to derive a priori expectations about relative entrainment strength across rhythm types. Within this context, the absence of a main effect may reflect a balance between these mechanisms under the present conditions. We therefore interpret the null effect cautiously and view it as an important direction for future work aimed at clarifying how rhythmic structure shapes neural tracking across sensory modalities.

## Behavioral precision and temporal complexity

The behavioral data converge with the neural findings. Deaf participants showed lower tapping variability than hearing controls, indicating more precise SMS to vibrotactile beats. Importantly, the Group × Rhythm interaction revealed that this advantage was driven by complex (“house”) rhythms, with no reliable group difference for the metronome stimuli (Figure 3). Metronomic patterns can be supported by relatively simple interval timing, whereas complex rhythms require extraction of a beat from higher-order temporal structure. The selective advantage for complex rhythms in the deaf group therefore suggests that their enhancement is not limited to basic interval tracking, but extends to more sophisticated temporal prediction.

Although our task did not directly assess rhythm discrimination, the differential SMS performance across rhythms in the deaf group implies that participants were sensitive to differences in temporal structure at a perceptual level. In developmental work, successful discrimination of rhythmic patterns is often taken as a prerequisite for the emergence of higher-order beat sensitivity (Hannon et al. 2018). By analogy, the pattern of reduced variability for complex vibrotactile rhythms in deaf individuals is consistent with a more finely tuned representation of vibrotactile temporal structure, potentially reflecting long-term experience with complex vibrotactile music (eg in amplified social settings like dance clubs). From a Bayesian perspective, such experience may shape priors over vibrotactile rhythmic patterns, facilitating more efficient extraction of a beat from complex temporal input. Hearing individuals, who typically encounter vibrotactile stimulation as a by-product of auditory music, may have weaker or less specific priors for complex vibrotactile rhythms. This could help explain the dissociation between groups for complex (ie house rhythms), but not metronomic, stimuli.

## Brain x behavior relationship

Contrary to expectations, we did not observe a significant relationship between neural entrainment and tapping variability across groups. Although group differences were evident in both neural and behavioral measures, such that the deaf group exhibited heightened neural entrainment and more stable SMS, these indices did not covary at the individual level. This dissociation suggests that neural entrainment and SMS may reflect partially dissociable processes supporting beat perception. Neural entrainment has been theorized to reflect the alignment of endogenous oscillations with rhythmic structure, supporting temporal prediction and tracking (Large and Kolen 1994; Large and Snyder 2009), whereas SMS has been proposed to rely on sensorimotor integration and motor planning processes involving regions such as the supplementary motor area and premotor cortex (Repp and Su 2013).

One possible explanation for the present null finding is the mismatch in task demands: neural entrainment was measured during passive listening, whereas tapping variability reflects active synchronization, which engages additional predictive and motor control mechanisms. As such, neural tracking of rhythmic structure may not directly map onto motor performance when assessed in separate contexts. More broadly, these findings suggest that enhanced neural entrainment does not necessarily translate into more stable SMS at the individual level. Notably, the absence of a reliable association, coupled with relatively constrained variability in tapping performance, suggests that any relationship between these measures may be small in magnitude.

Future research on vibrotactile beat perception would benefit from assessing brain–behavior relationships within a unified task context, for example by recording EEG during active synchronization, and by examining coupling at finer temporal scales.

## Sensory and cognitive adaptations supporting vibrotactile beat sensitivity

Our results raise mechanistic questions about how temporal prediction is instantiated in the brains of deaf individuals. Although the present data do not permit direct localization of the underlying circuitry, they are consistent with multiple, partially overlapping adaptations: (i) cross-modal recruitment of auditory–motor networks, (ii) altered contributions from vestibular pathways, and (iii) changes in top-down attentional control. Below we outline how each of these mechanisms might contribute to the observed enhancements.

## Cross-modal recruitment of auditory–motor networks

A substantial literature demonstrates that deafness leads to cross-modal recruitment of auditory cortices by somatosensory and visual inputs (Levänen et al. 1998; Auer et al. 2007; Lomber et al. 2010; Karns et al. 2012). Vibrotactile stimulation can elicit robust responses in primary and secondary auditory regions in deaf adults, sometimes exceeding those evoked by visual stimulation (Karns et al. 2012). Animal work further shows increased cross-modal projections between auditory and somatosensory cortices after deafening (Allman et al. 2009; Meredith et al. 2012), suggesting structural as well as functional reorganization.

## Adaptations in top-down attention

A third class of explanations concerns top-down attentional control rather than (or in addition to) changes in early sensory coding. The dorsal attention network, encompassing posterior parietal and frontal regions, is known to be sensitive to sensory deprivation and cross-modal plasticity (Bavelier and Neville 2002; Bavelier et al. 2006; Dell Ducas et al. 2021). Enhancements in visual motion processing in deaf individuals have been linked to adaptations in posterior parietal cortex (Shiell et al. 2014), suggesting that attentional resources may be reallocated toward remaining modalities.

Analogously, the enhanced vibrotactile beat sensitivity observed here may reflect an increased capacity to allocate and sustain attention to temporal structure in vibrotactile input. This could manifest as more precise tracking of event onsets, improved maintenance of an internal beat representation, or more effective suppression of noise in the temporal signal. On this account, the group differences we observe would arise from adaptations in the deployment of attention within shared timing networks, rather than solely from changes at the level of primary sensory cortex.

## Possible indirect contributions from vestibular system

A second, more indirect consideration concerns the vestibular system, which has been implicated in rhythm and beat processing in movement-based contexts (Phillips-Silver and Trainor 2005, 2008; Trainor et al. 2009). However, the present paradigm was not designed to engage vestibular pathways directly. Vibrotactile stimulation was delivered to the torso and thighs at amplitudes unlikely to induce head acceleration sufficient to drive vestibular receptors, and participants were instructed to remain still throughout the task. Accordingly, we do not interpret the present findings as reflecting vestibular contributions to beat-based prediction.

More broadly, the vestibular system may play a role in rhythm perception under conditions involving whole-body movement (Trainor et al. 2009) or low-frequency stimulation (Cameron et al. 2022), but its contribution to vibrotactile beat processing in stationary contexts remains unclear. Future work using paradigms that explicitly manipulate head motion or vestibular input will be required to determine whether vestibular signals interact with somatosensory and auditory–motor networks in supporting temporal prediction.

These three mechanistic accounts—cross-modal recruitment of auditory–motor networks, attentional reallocation, and vestibular contributions—are not mutually exclusive. Together they provide a framework for interpreting vibrotactile beat sensitivity as an emergent property of a reconfigured timing network in deaf individuals. In this sense, the present findings resonate with the notion of “Deaf Gain” (Bauman and Murray 2009), emphasizing not only what is lost with auditory deprivation but also the emergence of distinct strengths in non-auditory temporal processing.

## Future directions

Disentangling the relative contributions of these mechanisms will require targeted experimental designs and multimodal imaging. One promising avenue is to combine frequency-tagging EEG with spatially resolved techniques such as fNIRS to localize vibrotactile beat-related activity within auditory, somatosensory, vestibular, and dorsal attention networks. Demonstrating that vibrotactile beat frequencies preferentially activate reorganized auditory cortices in deaf individuals would provide direct support for the cross-modal recruitment account.

A second direction is to probe vestibular involvement more directly, for example by parametrically manipulating very-low-frequency components, using head-coupled transducers, or applying galvanic vestibular stimulation (Fitzpatrick and Day 2004) in conjunction with vibrotactile rhythms. Such work could clarify whether vestibular signals contribute to beat sensitivity in deaf individuals and how they interact with somatosensory input.

Finally, the attentional account could be tested by incorporating tasks that explicitly tax sustained, selective, or divided attention to vibrotactile rhythms, and by relating performance to neural markers of top-down control. Comparing deaf and hearing participants on continuous performance tasks with vibrotactile temporal structure, or on dual-task paradigms that compete for attentional resources, would help determine whether enhanced beat sensitivity reflects changes in sensory representations, attentional deployment, or both.

## Limitations

Several limitations qualify the present findings. First, although SMS and neural entrainment are well-established markers of beat sensitivity, they do not capture all dimensions of rhythm perception, such as explicit beat awareness, metrical interpretation, or discrimination of closely related rhythmic patterns. Including vibrotactile rhythm discrimination, perturbation detection, or interval judgment tasks in future work would provide a more complete characterization of vibrotactile rhythm processing.

Second, the deaf sample was heterogeneous with respect to age of onset and severity of hearing loss, and showed greater variability in neural measures than the hearing group (Figure 2a). This heterogeneity likely reflects differences in language exposure, educational environments, music experience, and clinical history (Cannon et al. 2016; Smith and Allman 2020). Our sample size did not afford sufficient power to model these factors in detail, and exploratory analyses of the deafness subgroup should be interpreted cautiously. Future studies with larger samples and more fine-grained characterization of auditory, linguistic, and music experience will be essential for understanding individual differences.

Third, we did not collect detailed measures of music engagement or rhythmic experience, in part to avoid imposing hearing-centric assumptions about what constitutes “musical” activity. In retrospect, co-developed questionnaires with members of the Deaf community could provide culturally appropriate ways to quantify relevant experiential variables. Such measures would be particularly valuable for testing experience-based hypotheses about complex vibrotactile rhythms.

An additional limitation concerns the absence of an auditory control condition. As a result, we cannot directly compare the precision of vibrotactile beat-based prediction observed here to auditory-based temporal prediction in hearing individuals. Tranchant et al. (2017) suggest that auditory synchronization may be more precise than vibrotactile synchronization, even among individuals with extensive experience relying on tactile input. Accordingly, the present findings should be interpreted as demonstrating that vibrotactile input is sufficient, and in some cases enhanced in deaf individuals, without implying equivalence to auditory-based timing. Direct within-subject comparisons across sensory modalities will be an important direction for future research.

Finally, our whole-brain entrainment measure does not reveal the spatial distribution or laminar origin of beat-related responses, nor does it distinguish definitively between oscillatory and evoked components. While this is common to the frequency-tagging literature, future work combining scalp EEG with source localization or intracranial recordings would help refine the interpretation of these signals.

## Conclusions

The present study provides converging neural and behavioral evidence that beat-based temporal prediction can be robustly supported through vibrotactile input in the absence of auditory experience. Deaf individuals showed stronger frequency-tagged entrainment to vibrotactile beats and more precise SMS than hearing controls, with a selective advantage for complex rhythms that require higher-order temporal integration. These findings suggest that timing networks typically engaged by auditory rhythms can be accessed via somatosensory pathways after auditory deprivation. More broadly, they illustrate how cross-modal plasticity can give rise to enhanced non-auditory temporal processing, offering a mechanistic perspective on aspects of musical rhythm perception for deaf individuals.
